# Supplementary material for: Genome-wide detection of imprinted differentially methylated regions using nanopore sequencing
Source: eLife. 2022 Jul 5;11:e77898. doi: 10.7554/eLife.77898 (PMC9255983; doi:10.7554/eLife.77898)
Supplement: Transparent reporting form [file elife-77898-transrepform1.pdf]

Westbrook Centre
Milton Road
Cambridge CB4 1YG, UK

P 01223 855340
W elifesciences.org
T @elife

eLife's transparent reporting form

We encourage authors to provide detailed information *within their submission* to facilitate the interpretation and replication of experiments. Authors can upload supporting documentation to indicate the use of appropriate reporting guidelines for health-related research (see EQUATOR Network), life science research (see the BioSharing Information Resource), or the ARRIVE guidelines for reporting work involving animal research. Where applicable, authors should refer to any relevant reporting standards documents in this form.
If you have any questions, please consult our Journal Policies and/or contact us: editorial@elifesciences.org.

Sample-size estimation

- You should state whether an appropriate sample size was computed when the study was being designed
- You should state the statistical method of sample size computation and any required assumptions
- If no explicit power analysis was used, you should describe how you decided what sample (replicate) size (number) to use

Please outline where this information can be found within the submission (e.g., sections or figure legends), or explain why this information doesn't apply to your submission:

No explicit power analysis was used.

We used 12 B-lymphocyte cell lines nanopore public data with their parental information in 1000 genome project and genome-in-a-bottle. This information can be found under materials and methods section "Nanopore Sequencing Data and Detection of Allele-Specific Methylation".

We used public whole-genome bisulfite sequencing (WGBS) data to confirm nanopore results, including 60 WGBS data for 29 tissue type samples from the Epigenomics Roadmap and ENCODE projects and 119 blood WGBS datasets for 87 individuals from the Blueprint project. This information can be found under materials and methods section "WGBS Data and Detection of Novel DMRs".

We used WGBS data from 1 blastocyst, 3 sperm and 2 oocyte libraries and 3 fetal tissue types to investigate germline or somatic origin of detected DMRs. This information can be found under materials and methods section "Detection of Germline and Somatic DMRs".

We used 16 WGBS datasets from mice, 34 WGBS datasets for rhesus macaque, and 22 WGBS datasets for chimpanzee to investigate the conservation of imprinting in the ortholog regions. We also used 2 embryo, 1 sperm, and 3 oocyte WGBS libraries in mouse and 1 embryo, 1 sperm, and 1 oocyte WGBS libraries in rhesus macaque to investigate germline or somatic origin of imprinted ortholog intervals. This information can be found under materials and methods section "Mammalian Conservation of DMRs".

We used ChIP-seq data for 6 B-lymphocyte cell lines including NA12878, NA12891, NA12892, NA19238, NA19239, and NA19240 to investigate allelic histone marks. This information can be found under materials and methods section "Allelic H3K4me3, H3K36me3, and H3K27me3 Analysis".

Replicates

- You should report how often each experiment was performed
- You should include a definition of biological versus technical replication
- The data obtained should be provided and sufficient information should be provided to indicate the number of independent biological and/or technical replicates
- If you encountered any outliers, you should describe how these were handled
- Criteria for exclusion/inclusion of data should be clearly stated
- High-throughput sequence data should be uploaded before submission, with a private link for reviewers provided (these are available from both GEO and ArrayExpress)

Please outline where this information can be found within the submission (e.g., sections or figure legends), or explain why this information doesn't apply to your submission:

This information doesn't apply to our submission.<br>We have used datasets for a lot of independent samples for each goal where no additional replicates were needed.

eLife Sciences Publications, Ltd is a limited liability non-profit non-stock corporation incorporated in the State of Delaware, USA, with company number 5030732, and is registered in the UK with company number FC030576 and branch number BR015634 at the address Westbrook Centre, Milton Road, Cambridge CB4 1YG.

Statistical reporting

- Statistical analysis methods should be described and justified
- Raw data should be presented in figures whenever informative to do so (typically when N per group is less than 10)
- For each experiment, you should identify the statistical tests used, exact values of N, definitions of center, methods of multiple test correction, and dispersion and precision measures (e.g., mean, median, SD, SEM, confidence intervals; and, for the major substantive results, a measure of effect size (e.g., Pearson's r, Cohen's d)
- Report exact p-values wherever possible alongside the summary statistics and 95% confidence intervals. These should be reported for all key questions and not only when the p-value is less than 0.05.

Please outline where this information can be found within the submission (e.g., sections or figure legends), or explain why this information doesn’t apply to your submission:

All the statistical tests, p-values and other related metrics are reported under appropriate sections.

Statistical analysis for detection of DMRs from nanopore data can be find under result section “Assessing the Effectiveness of Nanopore Methylation Calling and Detection of Known Imprinted DMRs” and materials and methods section “Nanopore Sequencing Data and Detection of Allele-Specific Methylation”.

Statistical analysis for the validation of nanopore results using WGBS data can be find under result section “Confirmation of Novel Imprinted DMRs” and materials and methods section “WGBS Data and Detection of Novel DMRs ".

Statistical analysis for determination of germline and somatic DMRs using WGBS data can be find under result section “Determination of Germline versus Somatic Status of Novel Imprinted DMRs” and materials and methods section “Detection of Germline and Somatic DMRs ".

Statistical analysis for determination of allelic H3K4me3, H3K36me3, and H3K27me3 histone marks using ChIP-seq data can be find under the result sections “Allelic H3K4me3 Histone Mark at Detected DMRs” and “Enriched Allelic H3K36me3 and H3K27me3 Histone Marks at Contiguous Blocks” and under materials and methods section “Allelic H3K4me3, H3K36me3, and H3K27me3 Analysis".

Statistical analysis for investigation of conservation using WGBS data for mus musculus, rhesus macaque, and chimpanzee can be found under result section “Conservation of Detected Imprinted DMRs across Mammals” and materials and methods section “Mammalian Conservation of DMRs".

(For large datasets, or papers with a very large number of statistical tests, you may upload a single table file with tests, Ns, etc., with reference to sections in the manuscript.)

Group allocation

- Indicate how samples were allocated into experimental groups (in the case of clinical studies, please specify allocation to treatment method); if randomization was used, please also state if restricted randomization was applied

eLife Sciences Publications, Ltd is a limited liability non-profit non-stock corporation incorporated in the State of Delaware, USA, with company number 5030732, and is registered in the UK with company number FC030576 and branch number BR015634 at the address Westbrook Centre, Milton Road, Cambridge CB4 1YG.

- Indicate if masking was used during group allocation, data collection and/or data analysis

Please outline where this information can be found within the submission (e.g., sections or figure legends), or explain why this information doesn't apply to your submission:

Not applicable to our study because it is not a clinical study.

Additional data files ("source data")

- We encourage you to upload relevant additional data files, such as numerical data that are represented as a graph in a figure, or as a summary table
- Where provided, these should be in the most useful format, and they can be uploaded as "Source data" files linked to a main figure or table
- Include model definition files including the full list of parameters used
- Include code used for data analysis (e.g., R, MatLab)
- Avoid stating that data files are "available upon request"

Please indicate the figures or tables for which source data files have been provided:

Figure 2 - source data 1 is the source data for Figure 2a<br>Figure 2 - source data 2 is the source data for Figure 2b<br>Appendix 1 - figure 1 - source data 1 is the source data for Appendix 1 - figure 1<br>Figure 4 - source data 1 is the source data for Figure 4a<br>Figure 4 - source data 2 is the source data for Figure 4b<br>Figure 9 - source data 1 is the source data for Figure 9<br>Figure 9 - figure supplement 1 - source data 1 is the source data for Figure 9 - figure supplement 1
